# Case Report: IVIG as a bridging strategy in high-risk antiphospholipid syndrome with infected cutaneous ulceration and thrombocytopenia

**DOI:** 10.3389/fimmu.2026.1867840

**Published:** 2026-07-28

**Authors:** Aliaa Khalili, Nouraldeen Deeb, Salahaldeen Deeb, Fares Sayed Ahmed, Younis Malik Younis Amro, Abdallah Altell

**Affiliations:** Faculty of Medicine, Al-Quds University, Jerusalem, Palestine

**Keywords:** antiphospholipid syndrome, bridging therapy, cutaneous ulceration, intravenous immunoglobulin, management strategy, thrombocytopenia

## Abstract

**Background:**

Severe cutaneous ulceration in antiphospholipid syndrome (APS) with concurrent infection and thrombocytopenia presents a therapeutic dilemma: standard immunosuppression risks worsening infection, while anticoagulation risks hemorrhage. We present a high-risk case successfully managed using intravenous immunoglobulin (IVIG) as a bridging immunomodulatory strategy.

**Case presentation:**

A 73-year-old man with triple-positive APS and immune thrombocytopenic purpura (ITP) developed rapidly progressive leg ulcers with clinical features consistent with pyoderma gangrenosum-like disease, complicated by active MRSA and *Pseudomonas aeruginosa* infection and severe thrombocytopenia (12 × 10^9^/L). Standard therapies were contraindicated: corticosteroids risked worsening infection; anticoagulation risked hemorrhage. The patient received IVIG (0.4 g/kg per dose, every 3 weeks) as primary immunomodulation, concurrent targeted antimicrobial therapy, temporary anticoagulation interruption, and discontinuation of baseline mycophenolate mofetil. Following platelet recovery above 70 × 10^9^/L, therapeutic anticoagulation was resumed with enoxaparin (80 mg subcutaneously twice daily) and aspirin (75 mg daily). Over five months, progressive re-epithelialization culminated in complete healing with platelet recovery.

**Conclusions:**

This case supports the feasibility of IVIG as a bridging immunomodulatory strategy in high-risk APS cutaneous disease where standard therapies are contraindicated by concurrent infection and thrombocytopenia. The sequential management approach successfully resolved a clinically challenging scenario. This case illustrates how risk-adapted immunomodulation may enable safe management of complex APS manifestations.

## Introduction

Cutaneous manifestations occur in approximately 49% of patients with antiphospholipid syndrome (APS), ranging from livedo racemosa to digital gangrene and chronic leg ulcers (1). While these lesions classically reflect aPL-mediated microvascular thrombosis, emerging evidence suggests that some APS-associated ulcers may present with inflammatory features, raising diagnostic and therapeutic challenges (2–5).

A particular clinical challenge arises when APS cutaneous disease is complicated by concurrent infection and severe thrombocytopenia. In such scenarios, standard therapeutic approaches become contraindicated: high-dose corticosteroids (first-line for neutrophilic dermatoses) risk exacerbating active infection, while anticoagulation risks hemorrhage. Intravenous immunoglobulin (IVIG) offers a potential solution—providing immunomodulation without broad immunosuppression—but its role in this specific context remains poorly defined.

We present a complex case of a destructive leg ulcer in a patient with triple-positive APS and severe ITP that was managed with IVIG as a primary immunomodulatory intervention in a high-risk setting where standard therapies were contraindicated. By detailing the management approach and clinical outcomes, we aim to illustrate how IVIG may serve as a bridging strategy in this challenging clinical scenario.

## Case presentation

A 73-year-old Middle Eastern male with established triple-positive APS and ITP presented with non-healing skin ulcers on his right lower leg. His medical history included hypertension and atrial fibrillation. Baseline medications included apixaban (5 mg twice daily) for stroke prevention in atrial fibrillation and mycophenolate mofetil (500 mg twice daily) for immunomodulation. He had received multiple prior courses of systemic corticosteroids for ITP management, but had not been on chronic corticosteroid therapy at the time of ulcer presentation. Two months prior to ulcer onset, he underwent an uneventful parotidectomy for a benign adenomatous tumor.

In May 2025, a small, painful ulcer appeared on his right anterior shin without preceding trauma. Despite routine wound care, the lesion enlarged and multiplied. By July 2025, examination revealed three ulcers on the right lower limb: two with partial granulation and one enlarging lesion with a necrotic base and undermined, violaceous borders—features clinically consistent with PG-like ulceration. Peripheral pulses were intact, and Doppler ultrasound confirmed normal venous and arterial flow, excluding ischemic and thrombotic etiologies.

Laboratory investigations revealed a persistently prolonged activated partial thromboplastin time (aPTT) and severe thrombocytopenia (12 × 10^9^/L). C-reactive protein was mildly elevated at 3.8 mg/L (normal <6 mg/L). Wound cultures grew methicillin-resistant *Staphylococcus aureus* (MRSA) initially, and subsequently *Pseudomonas aeruginosa*.

### Diagnostic considerations

The clinical morphology of the enlarging ulcer with necrotic center and undermined borders raised the differential diagnosis of PG-like ulceration; however, other etiologies including APS-related ischemic ulcer with secondary infection, vasculitic ulcer, or infectious ulcer mimicking PG could not be excluded. Skin biopsy, which would normally be performed to confirm the diagnosis, was deferred due to two significant risks: (1) the high likelihood of pathergy (trauma-induced ulcer exacerbation), and (2) the prohibitive bleeding risk posed by severe thrombocytopenia. Consequently, diagnostic classification remains uncertain, and the ulcer is best described as a neutrophilic-appearing inflammatory ulcer of uncertain etiology (**Table 1**).

**Table 1:** Clinical and diagnostic features of the inflammatory ulcer.

Feature	Present	Notes
Rapid progression	✓	Ulcers enlarged over weeks
Violaceous, undermined borders	✓	Characteristic appearance noted
Severe pain	✓	Patient reported significant pain
Exclusion of infection	×	MRSA and *Pseudomonas* cultured from wound
Exclusion of vasculitis	✓	Normal vascular flow on Doppler
Exclusion of malignancy	✓	No evidence of malignancy

The presence of active bacterial infection (MRSA and Pseudomonas aeruginosa) complicates diagnostic classification and raises the possibility that infection was the primary pathologic driver, with inflammatory features potentially representing a secondary response to bacterial invasion.

Diagnostic uncertainty cannot be resolved without histologic confirmation. The ulcer may represent true pyoderma gangrenosum, infection-driven ulceration with secondary inflammation, or APS-related ischemia with inflammatory response.

### Management strategy

The clinical scenario presented competing risks: active bacterial infections contraindicated high-dose systemic corticosteroids [the first-line therapy for PG (6)], while severe thrombocytopenia and active wound bleeding necessitated temporary interruption of anticoagulation. Baseline immunosuppression with mycophenolate mofetil was discontinued to facilitate infection clearance.

The multidisciplinary team initiated IVIG (0.4 g/kg per dose, administered intravenously, repeated every 3 weeks) as a primary immunomodulatory intervention. This approach was selected because IVIG: (1) provides immunomodulation without broad immunosuppression that might exacerbate active infections; (2) addresses severe ITP through multiple mechanisms (Fc receptor blockade, anti-idiotypic antibodies, FcRn saturation); and (3) has documented efficacy in refractory inflammatory ulcers (7). Alternative immunosuppressive agents such as ciclosporin or systemic corticosteroids were avoided due to the risk of worsening active bacterial infections. The dosing regimen of 0.4 g/kg per dose every 3 weeks was chosen to balance immunomodulatory efficacy with practical feasibility in an outpatient setting.

Concurrently, the patient received targeted systemic antimicrobial therapy (anti-MRSA and anti-Pseudomonas agents) and optimized local wound care. DOACs (apixaban) were discontinued due to active wound bleeding and severe thrombocytopenia. Temporary anticoagulation interruption was guided by severe thrombocytopenia (12 × 10^9^/L) and active bleeding risk, with the understanding that resumption would be prioritized once platelet recovery was achieved. Once platelet counts recovered above 70 × 10^9^/L, therapeutic anticoagulation was resumed with enoxaparin (80 mg subcutaneously twice daily, weight-adjusted) and antiplatelet therapy with aspirin (75 mg daily) was initiated.

### Clinical course and imaging

**Figure 1:**
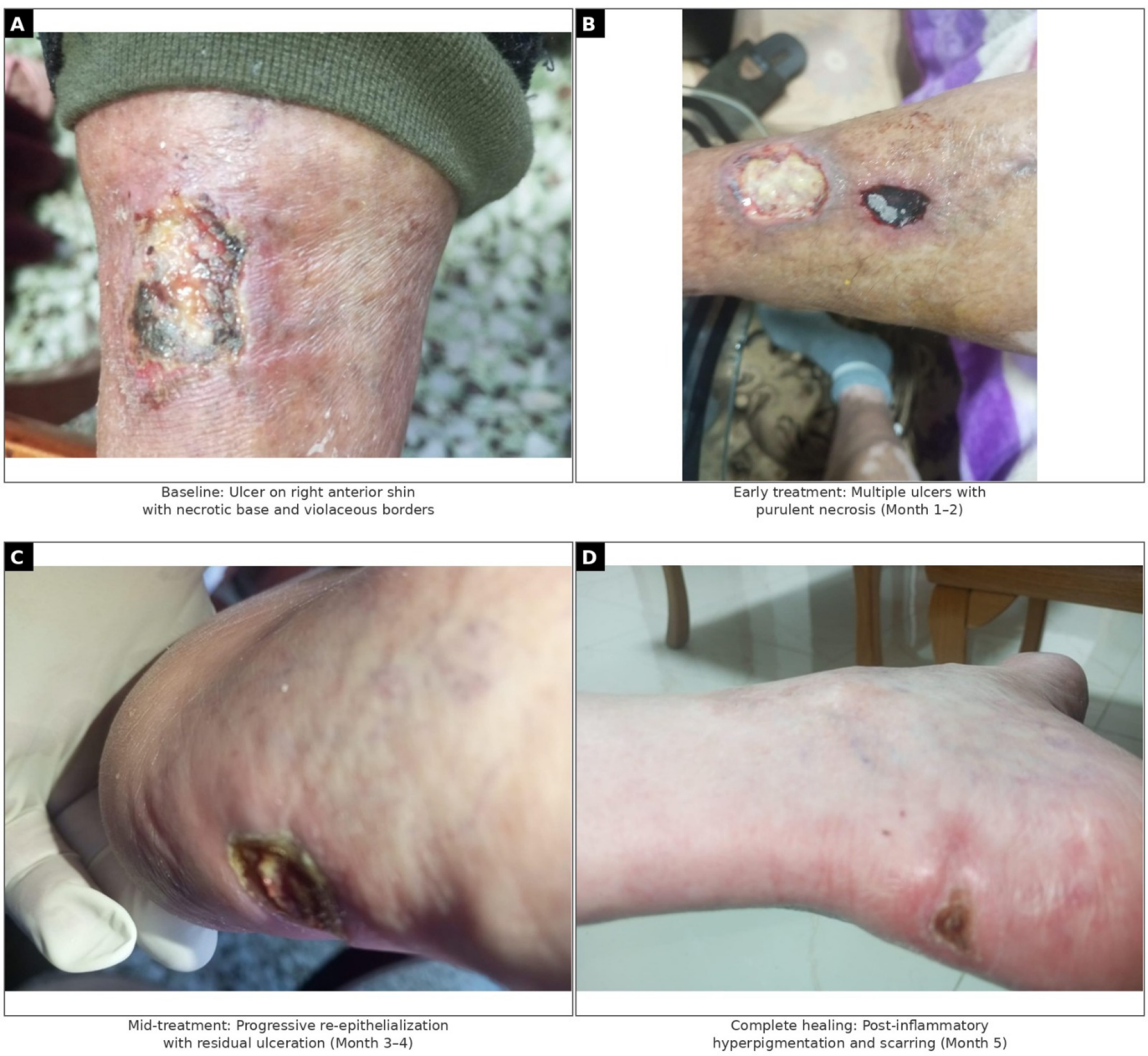
Clinical progression of APS-associated cutaneous ulceration. Sequential photographs documenting the five-month clinical course from initial presentation through complete healing. **(A)** Early stage (Month 1): Small, painful ulcer with minimal surrounding erythema on the right anterior shin. **(B)** Active phase (Month 2): Multiple ulcers with characteristic necrotic centers and undermined, violaceous borders consistent with pyoderma gangrenosum-like morphology. Note the dark necrotic base and surrounding erythematous halo. **(C)** Intermediate stage (Month 3): Progressive resolution with formation of granulation tissue, reduced necrosis, and evidence of wound bed preparation. Surrounding erythema remains but borders are less undermined. **(D)** Healed phase (Month 5): Complete re-epithelialization with post-inflammatory hyperpigmentation and residual scarring. The ulcer bed is fully epithelialized with minimal surrounding inflammation. These images illustrate the clinical response to multifactorial intervention including targeted antimicrobial therapy, IVIG administration, and optimized wound care.

Over five months, the patient demonstrated steady clinical improvement with concurrent recovery of platelet counts and resumption of anticoagulation. Sequential photographic documentation revealed progressive resolution of the ulcers (**Figure 1**). Purulent necrosis resolved, and the ulcers progressively re-epithelialized, culminating in complete healing with post-inflammatory hyperpigmentation and scarring (**Figure 1**). No new lesions developed.

Hematologically, the platelet count recovered from 12 × 10^9^/L to 133 × 10^9^/L. Follow-up serologic testing performed four weeks after the final IVIG cycle revealed apparent seronegativity of the aPL profile, with lupus anticoagulant, anticardiolipin, and anti-β2-glycoprotein I antibodies all returning negative results (**Table 2**). This apparent seronegativity is likely influenced by IVIG administration or represents transient suppression rather than true remission, and requires confirmation with repeat testing ≥12 weeks later to exclude assay interference or transient effects.

**Table 2:** Clinical and laboratory parameters at baseline and follow-up.

Parameter	Baseline	Follow-up (Month 5)
Clinical Status	Active, enlarging necrotic ulcers	Complete re-epithelialization with scarring
Platelet Count (× 10^9^/L)	12	133
aPTT (seconds)	Prolonged (LA+)	Normalized (LA−)
Lupus Anticoagulant	Positive	Negative
Anticardiolipin IgG/IgM	Positive	Negative
Anti-β2GPI IgG/IgM	Positive	Negative
Anticoagulation Status	Apixaban (held due to bleeding)	Enoxaparin 80 mg SC BID + Aspirin 75 mg daily

aPTT, activated partial thromboplastin time; LA, lupus anticoagulant; IgG, immunoglobulin G; IgM, immunoglobulin M; β2GPI, beta-2 glycoprotein I.

## Discussion

### IVIG as a bridging immunomodulatory strategy

This case illustrates the clinical utility of IVIG in a high-risk scenario where standard therapeutic approaches were contraindicated. The patient presented with three competing clinical demands: (1) active bacterial infection requiring antimicrobial therapy and avoiding broad immunosuppression; (2) severe thrombocytopenia with active bleeding necessitating temporary anticoagulation interruption; and (3) rapidly progressive cutaneous ulceration requiring immunomodulation. IVIG addressed all three challenges simultaneously: it provided immunomodulation without broad immunosuppression, corrected severe ITP (enabling platelet-dependent hemostasis and eventual anticoagulation resumption), and avoided the infection-exacerbating effects of corticosteroids.

The mechanisms by which IVIG may have contributed to clinical improvement include Fc-gamma receptor (FcγR) blockade on innate immune cells, suppression of pro-inflammatory cytokines (IL-8, TNF-α), and neutralization of pathogenic autoantibodies via anti-idiotypic networks (8). However, in a single case with multiple confounding interventions, these mechanisms remain speculative.

### Pathophysiologic context: immunothrombosis in APS

This case can be interpreted within emerging immunothrombotic models of APS, which describe overlapping thrombotic and inflammatory mechanisms. Recent evidence demonstrates that aPL antibodies simultaneously activate coagulation and innate immune pathways, including neutrophil extracellular trap formation through complement-dependent mechanisms (3, 9–11). This bidirectional link between thrombosis and autoinflammation may explain why some APS patients present with inflammatory-appearing cutaneous ulcers rather than purely ischemic lesions. However, in this case, the presence of active bacterial infection complicates mechanistic interpretation, as infection itself may have been the primary driver of the inflammatory response.

### Confounding variables in clinical improvement

While complete healing occurred during the period of IVIG administration, attributing this solely to IVIG would be an oversimplification. The clinical improvement must be interpreted in the context of multiple simultaneous interventions. IVIG’s benefit may have been mediated in part through platelet recovery:

1. Targeted Antimicrobial Therapy: Eradication of MRSA and *Pseudomonas aeruginosa* likely reduced the local inflammatory burden and removed a significant barrier to wound healing. Antimicrobial therapy may have been a critical intervention if infection was the primary pathologic driver. The apparent PG-like morphology may have been a secondary response to bacterial invasion rather than true PG.
2. Platelet Recovery via IVIG: IVIG’s primary effect in this case may have been correction of severe ITP (platelet count recovery from 12 to 133 × 10^9^/L), which enabled: (a) resumption of therapeutic anticoagulation (enoxaparin), and (b) reduced active wound bleeding, thereby stabilizing the ulcer bed. However, direct anti-inflammatory effects on the ulcer cannot be definitively established.
3. Withdrawal of Mycophenolate Mofetil: Paradoxically, cessation of baseline immunosuppression may have facilitated infection clearance by allowing enhanced innate immune responses.
4. Anticoagulation Management: Initial interruption of DOACs reduced active wound bleeding and stabilized the ulcer bed during the acute phase. Subsequent resumption of therapeutic anticoagulation with enoxaparin (once platelet counts recovered above 70 × 10^9^/L) and addition of antiplatelet therapy (aspirin 75 mg daily) addressed the underlying thrombotic risk while maintaining hemostasis.
5. Optimized Wound Care: Consistent local management over five months is an independent, critical factor in chronic ulcer healing.

In this case, the most clinically actionable contribution of IVIG may have been hematologic stabilization through platelet recovery, which permitted reintroduction of therapeutic anticoagulation—a pivotal step in managing APS-related thrombotic risk. IVIG also provided immunomodulation without broad immunosuppression in the setting of active infection. In a single case with multiple confounders, the relative contribution of each intervention cannot be precisely determined.

## Limitations

This case report has several important limitations that constrain the strength of conclusions:

1. Lack of Histologic Confirmation and Active Bacterial Infection: Biopsy was not performed, so the diagnosis of PG remains “probable” based on clinical criteria alone. Critically, active bacterial infection (MRSA and *Pseudomonas aeruginosa*) was present, which is an exclusion criterion for definite PG diagnosis per Maverakis consensus criteria. This creates fundamental diagnostic uncertainty that cannot be resolved. We cannot distinguish between true PG, infection-driven ulceration, or APS-related ischemia with secondary inflammation. The histopathologic features (neutrophilic infiltration, abscess formation, vascular involvement) that would definitively classify the ulcer remain unknown. The presence of active infection substantially weakens any claim that this represents true PG.
2. Absence of Mechanistic Data: No NET markers (citrullinated histone H3, cell-free DNA), cytokine measurements, or immunophenotyping were performed. Therefore, the proposed immunothrombotic mechanisms remain theoretical and cannot be validated in this specific patient.
3. Multiple Confounding Interventions: The simultaneous administration of targeted antibiotics, cessation of mycophenolate, interruption of anticoagulation, and IVIG precludes precise attribution of efficacy to any single intervention. Antimicrobial therapy may have been a critical intervention if infection was the primary pathologic driver. IVIG’s benefit may have been mediated in part through platelet recovery, enabling resumption of anticoagulation and reduced wound bleeding.
4. Single Case: This is a single case report without external validation or control group. The findings are not generalizable and should be considered hypothesis-generating only. PG diagnosis may be overdiagnosed in this case; the ulcer may have represented infection-driven ulceration with secondary inflammation rather than true PG.
5. Short Follow-up for aPL Seroreversion: The apparent seroreversion was documented only four weeks after the final IVIG cycle. This timeframe is insufficient to exclude assay interference or transient suppression. Longer-term follow-up testing (3–6 months post-IVIG) would be required to determine if this represents true remission.
6. Potential Assay Interference: High-dose IVIG can interfere with immunoassays. The apparent seroreversion may represent false-negative results rather than true immunologic remission.

### Clinical implications and take-home message

**Figure 2:**
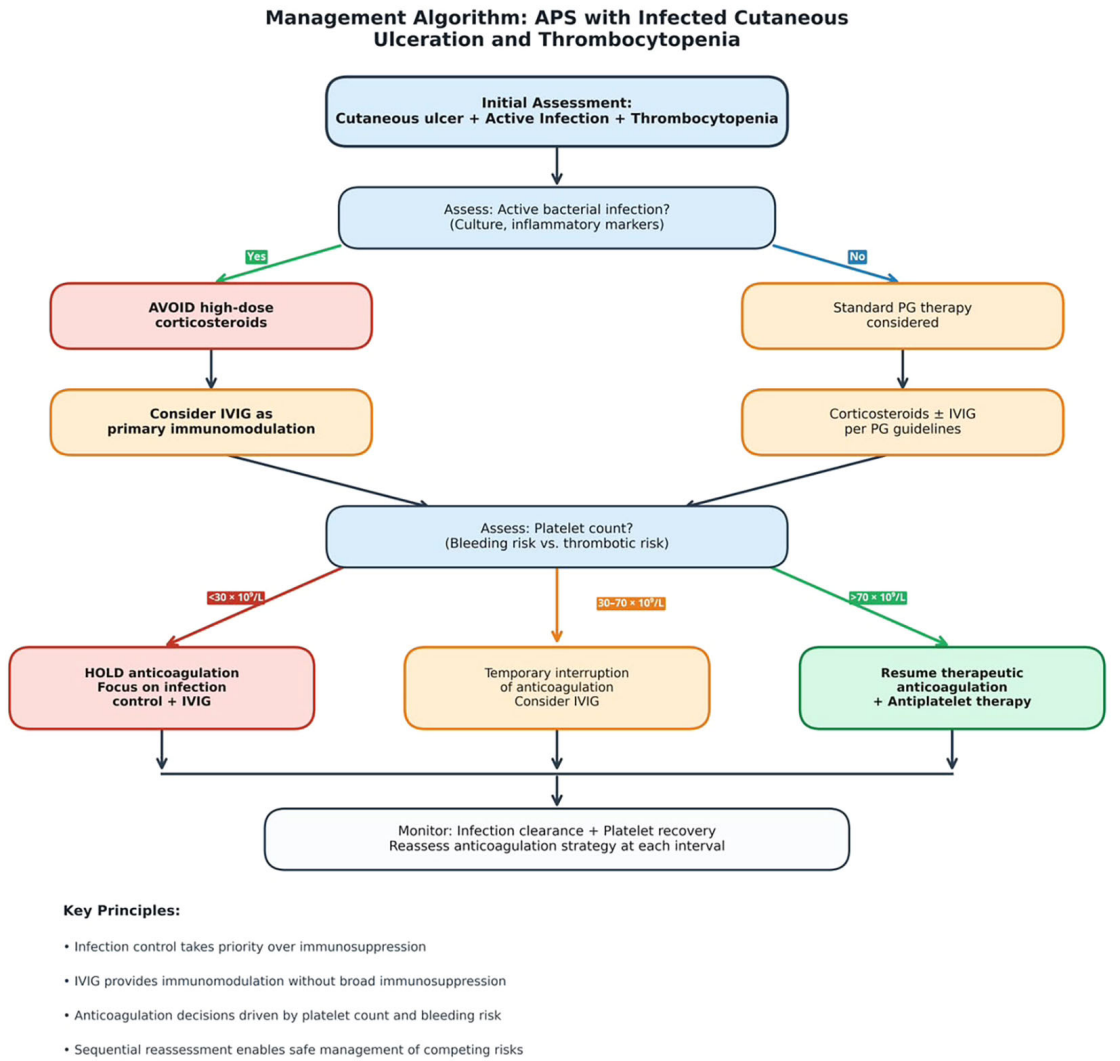
Proposed management algorithm for APS-associated cutaneous ulceration with concurrent infection and thrombocytopenia. Illustrative decision-making flowchart based on this case and existing clinical principles, not a formal guideline. The algorithm integrates three critical clinical parameters: presence of active bacterial infection, severity of thrombocytopenia, and bleeding versus thrombotic risk. It prioritizes infection control and avoidance of broad immunosuppression in the setting of active infection. IVIG is presented as a potential immunomodulatory strategy when corticosteroids are contraindicated. Anticoagulation decisions are driven by platelet count thresholds: hold anticoagulation if platelets <30 × 10^9^/L, consider temporary interruption if 30-70 × 10^9^/L, and resume therapeutic anticoagulation with antiplatelet therapy once platelets >70 × 10^9^/L. This framework is illustrative and requires validation in larger cohorts before clinical implementation.

This case demonstrates the feasibility of IVIG as a bridging immunomodulatory strategy in APS-associated cutaneous disease where standard therapies are contraindicated by concurrent infection and thrombocytopenia. In high-risk scenarios—particularly when active infections contraindicate broad immunosuppression and severe thrombocytopenia precludes immediate anticoagulation—IVIG may be considered as an infection-conscious, hematologically favorable immunomodulatory option. The sequential approach of temporary anticoagulation interruption followed by resumption of therapeutic anticoagulation (with enoxaparin) and addition of antiplatelet therapy (aspirin) once hematologic recovery is achieved was employed in this case and successfully resolved a clinically challenging scenario. A proposed management algorithm incorporating these principles is presented in **Figure 2**. Such decisions should be made within a multidisciplinary framework that integrates infection control, bleeding management, wound care optimization, and careful monitoring of hematologic parameters.

## Conclusion

This case illustrates how IVIG may serve as a bridging immunomodulatory strategy in high-risk APS cutaneous disease where standard therapies are contraindicated. While diagnostic classification remains uncertain and causality cannot be established due to multiple interventions, the sequential management approach successfully resolved a clinically challenging scenario. The case supports the feasibility of this risk-adapted approach in carefully selected patients with APS cutaneous ulceration complicated by concurrent infection and thrombocytopenia. Whether this approach is generalizable or represents optimal management requires further investigation. Larger studies with mechanistic characterization, histologic confirmation, and careful attribution of interventions are needed to establish optimal treatment strategies for this rare but clinically challenging overlap syndrome.

## Data Availability

The original contributions presented in the study are included in the article/supplementary material. Further inquiries can be directed to the corresponding author.
